# Guanine- 5-carboxylcytosine base pairs mimic mismatches during DNA replication

**DOI:** 10.1038/srep05220

**Published:** 2014-06-09

**Authors:** Toshihiro Shibutani, Shinsuke Ito, Mariko Toda, Rie Kanao, Leonard B. Collins, Marika Shibata, Miho Urabe, Haruhiko Koseki, Yuji Masuda, James A. Swenberg, Chikahide Masutani, Fumio Hanaoka, Shigenori Iwai, Isao Kuraoka

**Affiliations:** 1 Graduate School of Engineering Science, Osaka University Graduate School of Engineering Science, 1-3 Machikaneyama, Toyonaka, Osaka 560-8531 Japan; 2 Laboratory for Developmental Genetics, RIKEN Center for Integrative Medical Sciences, Yokohama 230-0045, Japan; 3 Research Institute of Environmental Medicine, Nagoya University, Furo-cho, Chikusa-ku, Nagoya 464-8601, Japan; 4 Department of Environmental Sciences and Engineering, Gillings School of Global Public Health, University of North Carolina at Chapel Hill, Chapel Hill, NC 27599, USA; 5 Department of Toxicogenomics, Nagoya University Graduate School of Medicine, 65 Tsurumai-cho, Showa-ku, Nagoya 466-8550, Japan; 6 Faculty of Science, Gakushuin University, 1-5-1 Mejiro, Toshima-ku, Tokyo 171-8588, Japan

## Abstract

The genetic information encoded in genomes must be faithfully replicated and transmitted to daughter cells. The recent discovery of consecutive DNA conversions by TET family proteins of 5-methylcytosine into 5-hydroxymethylcytosine, 5-formylcytosine, and 5-carboxylcytosine (5caC) suggests these modified cytosines act as DNA lesions, which could threaten genome integrity. Here, we have shown that although 5caC pairs with guanine during DNA replication in vitro, G·5caC pairs stimulated DNA polymerase exonuclease activity and were recognized by the mismatch repair (MMR) proteins. Knockdown of thymine DNA glycosylase increased 5caC in genome, affected cell proliferation via MMR, indicating MMR is a novel reader for 5caC. These results suggest the epigenetic modification products of 5caC behave as DNA lesions.

DNA methylation at the C5 position of cytosine (5mC) in the context of CpG dinucleotides regulates gene expression, retrovirus silencing, X chromosome inactivation, and other functions in mammalian cells^1^. The enzymes responsible for this modification, i.e., DNA methyltransferases (DNMT1–3), are well characterized and are required for normal development in mice^2^. Although DNA methylation was previously assumed to be a stable epigenetic modification, recent discovery of the ten-eleven translocation (TET) family of DNA dioxygenases (TET1–3) has shown that the methyl group of 5mC can be modified to 5-hydroxymethylcytosine (5hmC), adding a layer of complexity to the epigenetic regulation of DNA methylation^3^,^4^,^5^. Several studies have developed methods for genome-wide mapping of 5hmC by using either 5hmC-specific antibodies^6^,^7^,^8^ or chemical labeling to enrich 5hmC-containing DNA^9^,^10^. More recently methods for mapping at a single-nucleotide resolution level were also reported^11^,^12^. Those study suggests a role for 5hmC in transcriptional activation and repression in a genomic context-dependent manner^6^,^7^,^13^. 5hmC is relatively stable and can be found in various mouse tissues and embryonic stem (ES) cells, although levels differ between cell types^14^,^15^; therefore, 5hmC is viewed as an epigenetic modification.

During DNA replication, maintenance DNMT1 maintains symmetric CpG methylation with high specificity on the unmethylated strand of a hemi-methylated CpG sequence, but not in a hemi-hydroxymethylated CpG sequence, which could lead to passive DNA methylation^16^,^17^. Alternatively, 5hmC can be converted to 5-formylcytosine (5fC) and 5-carboxylcytosine (5caC) via TET protein-mediated consecutive oxidations (Figure 1A)^18^,^19^. Biochemical analyses suggest thymine DNA glycosylase (TDG) excises 5fC and 5caC, thereby generating an apurinic/apyrimidinic site that is in turn processed by the base excision repair (BER) machinery, suggesting an active DNA demethylation pathway^20^. The importance of TDG in maintaining appropriate DNA methylation has been indicated by targeted *Tdg* allele disruptions in mice; these knockout mice exhibit aberrant DNA methylation in a subset of gene promoters and enhancers, coincident with dysregulated gene expression^21^,^22^. Unlike other DNA glycosylases required for the BER pathway, *Tdg* knockout is embryonic lethal in mice, despite leaving DNA repair largely intact. Thus, TDG is essential for proper embryonic development, in part due to its role in maintaining epigenetic stability during cell-lineage commitment.

Although 5fC and 5caC are assumed to be part of the DNA demethylation pathway and should therefore have short half-lives, substantial amounts of 5fC and 5caC are present in various mouse tissues and ES cells^19^. A recent study showed that 5fC and 5caC are enriched at gene regulatory elements in *Tdg*-deficient ES cells, suggesting the involvement of 5fC and 5caC in transcriptional regulation^23^,^24^. Other studies have suggested 5fC induces G·C to A·T transition mutations during DNA replication when DNA polymerase encounters 5fC on template-strand DNA^25^,^26^,^27^,^28^. When 5fC and 5caC behave as mutagenic bases, TET protein-mediated consecutive oxidations of 5mC and 5hmC leads to deleterious consequences such as predisposition to cancer or apoptosis due to the accumulation of genomic mutations, unless 5mC oxidation is coupled with efficient elimination of 5fC and 5caC. Here, we studied the activity of DNA polymerases on oligonucleotide templates containing specifically located epigenetic cytosine products that were oxidatively modified and found that 5caC forms G·T mismatch-mimicking base pairs with unmodified guanine. These mismatch-like base pairs induced DNA polymerase exonuclease activity and were recognized by mismatch repair (MMR) proteins, suggesting a novel DNA damage effect of 5caC via unexpected abortive MMR.

## Results

### DNA polymerases incorporat dGTP opposite modified cytosines

**Figure 1:**
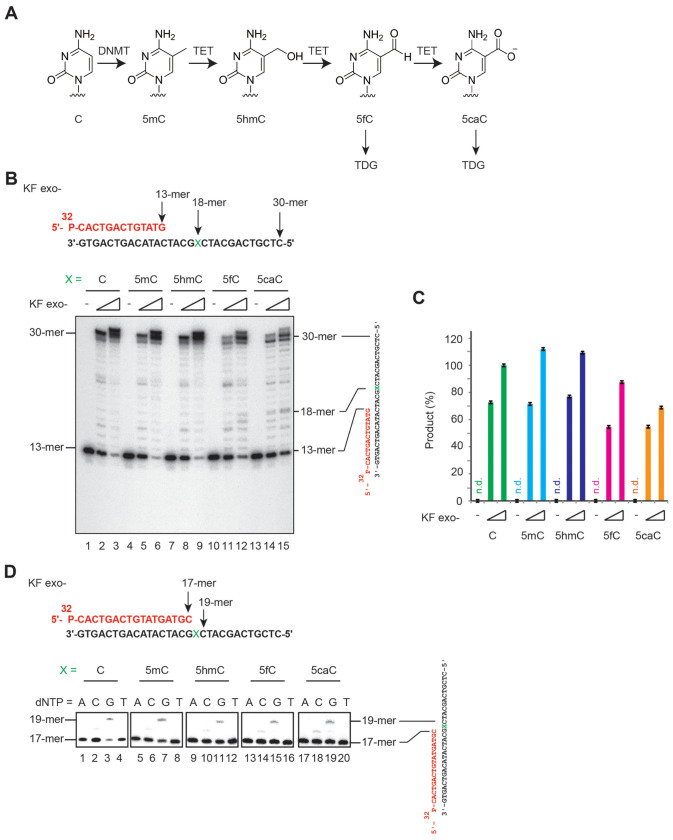
DNA synthesis by Klenow fragment exonuclease minus (KF exo-) on a DNA template containing 5fC and 5caC. (A) Proposed 5mC oxidation pathway involving TET dioxygenases. 5fC and 5caC are removed by TDG in the BER pathway. (B) A 13-mer primer was 5′-labeled with ^32^P and annealed with a 30-mer oligonucleotide containing the indicated modified cytosines at position X (upper panel). The primer/template complexes were incubated with increasing amounts of KF exo- (0, 0.04, and 1 U in each group of three lanes) at 30°C for 5 min. (C) Relative KF exo- DNA synthesis efficiency on all 5 modified cytosine templates. Data were normalized to the DNA synthesis efficiency of KF exo- (1 U) for normal cytosine-containing templates (lane 3). Quantification of the 30–31-nt fragments by image analysis. Error bars indicate the standard deviation. n.d.: not determined. (D) A 17-mer primer was 5′-labeled with ^32^P and was annealed with a 30-mer oligonucleotide containing the indicated modified cytosines at position X (upper panel). The primer/template complexes were incubated with KF exo- (0.01 U) for 5 min on ice with one of the indicated dNTPs (lanes 1–4, 5–8, 9–12, 13–16, and 17–20).

To assess whether these modified cytosines, which are involved in epigenetic processes, behave as DNA lesions that induce genomic mutations or block DNA synthesis, we first investigated whether DNA polymerases catalyze DNA synthesis on templates containing a site-specific C, 5mC, 5hmC, 5fC, or 5caC. Figures 1B and 1C show that Klenow fragment exonuclease minus (KF exo-) synthesized ~30-mer DNA fragments on templates containing C, 5mC, or 5hmC, but synthesized fewer ~30-mer DNA fragments on templates containing 5fC or 5caC. In the case of 5caC, a small fraction of polymerases stalled briefly at the modified cytosine. The results suggest that DNA synthesis by KF exo-, unlike the typical stalling induced by DNA damage, was affected by 5fC and 5caC. Next, we examined the nucleotide preference for incorporation opposite a modified cytosine by KF exo-. The polymerase preferentially incorporated dGTP opposite modified cytosines, suggesting these cytosine derivatives were not highly mutagenic and that the less-efficient DNA synthesis observed in the case of 5fC and 5caC was not due to miscoding (Figure 1D). Human DNA polymerase η (Polη) permits replication past DNA lesions on templates^29^,^30^; Polη exhibited a similar difference in DNA synthesis efficiency (Figure S1A and S1B) and preferentially incorporated dGTP opposite modified cytosines (Figure S1C).

### 5caC pairing with guanine stimulates the proofreading function of Polδ

**Figure 2:**
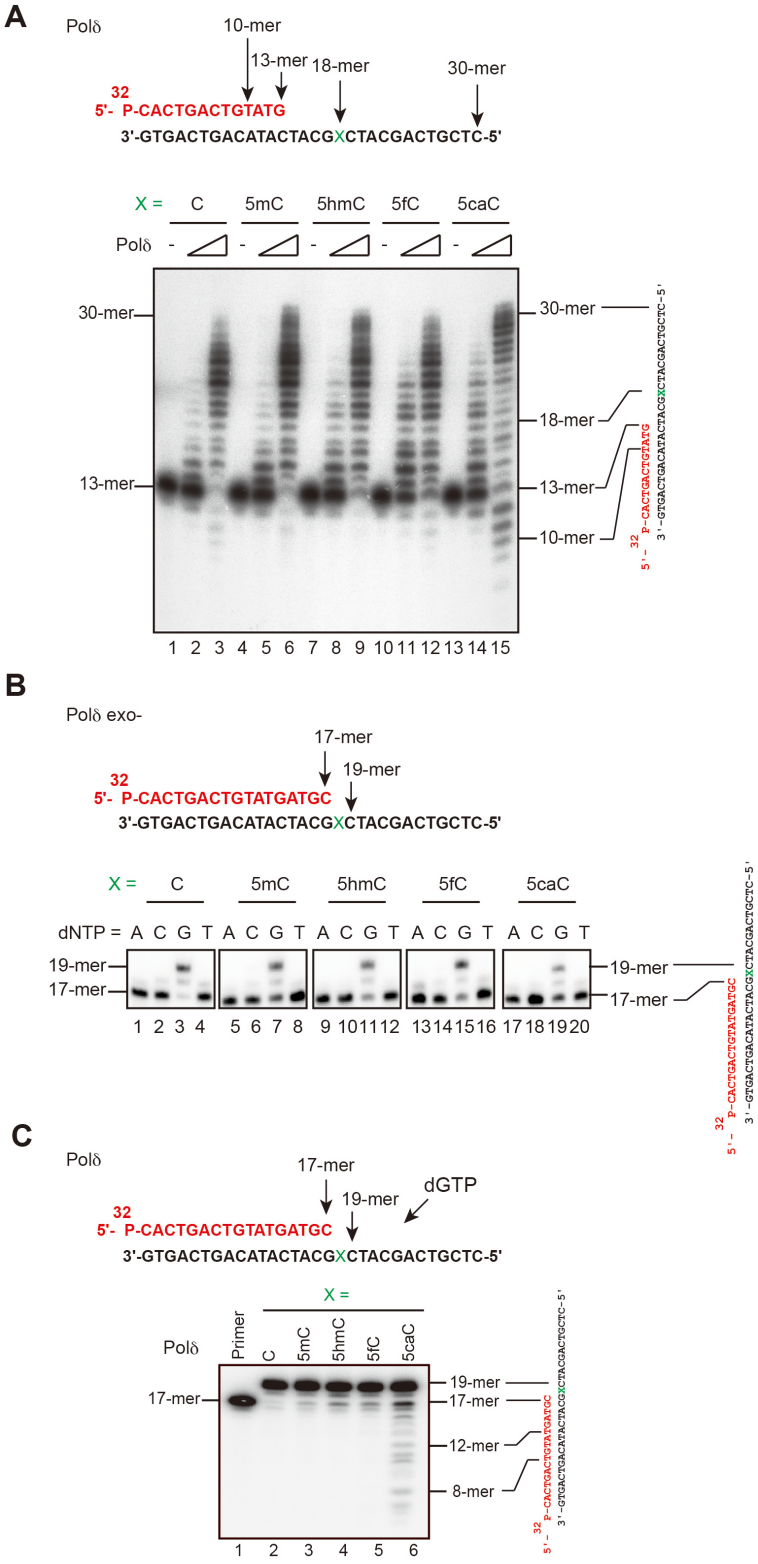
DNA synthesis reactions of human Polδ on DNA templates containing 5caC. (A) A 13-mer primer was 5′-labeled with ^32^P and annealed with a 30-mer oligonucleotide containing the indicated modified cytosines at position X (upper panel). The primer/template complexes were incubated (A) with increasing amounts of Polδ (0, 0.8, and 4 nM in each group of three lanes) at 30°C for 5 min. (B and C) A 17-mer primer was 5′-labeled with ^32^P and annealed with a 30-mer oligonucleotide containing the indicated modified cytosines at position X (upper panel). (B) The primer/template complexes were incubated with Polδ exo- (10 nM) for 5 min at 30°C with one of the indicated dNTPs (lanes 1–4, 5–8, 9–12, 13–16, and 17–20). (C) The 5′-labeled 17-mer primer was loading in the first lane. The primer/template complexes were incubated with pol δ (4 nM) and dGTP (100 μM) at 30°C for 5 min.

Both KF exo- and Polη were incapable of proofreading during DNA synthesis; therefore, we investigated whether human DNA polymerase δ (Polδ), which harbors an intrinsic 3′ to 5′ exonuclease domain, catalyzes DNA synthesis past the modified cytosines during replication. Interestingly, although Polδ synthesized DNA fragments on all templates, proofreading cleavage products were observed with only the 5caC templates (Figure 2A). Next, we examined the nucleotide preference for incorporation opposite a modified cytosine by Polδ exo-, which catalyzes DNA synthesis (Figure S2) and lacks 3′ to 5′ exonuclease activity. As shown in Figure 2B, this polymerase also incorporated dGTP opposite modified cytosines.

When base-pair misincorporation occurs during DNA synthesis, the proofreading exonuclease activity of DNA polymerase removes the incorrect base. To test whether this exonuclease activity ensured correct pairing of 5caC, polymerization reactions by Polδ were performed in the presence of only a single dGTP. Fragment degradation by the exonuclease activity of Polδ was simultaneously observed on the 5caC templates (Figure 2C), indicating that 5caC pairing with guanine stimulates the proofreading function of Polδ.

We observed similar DNA synthesis or exonuclease effects for 5caC during DNA synthesis by Klenow fragment (KF exo+), which possesses 3′ to 5′ exonuclease activity during DNA synthesis (Figure S3). KF exo+ possesses intrinsic terminal-deoxynucleotidyl transferase activity; therefore, it could synthesize DNA fragments up to approximately 20 bp on all templates (Figure S3C). In the case of KF exo+, primers annealed with 5caC templates were more degraded than those annealed with 5fC templates, indicating that G·5caC pairings stimulate the proofreading function more than G·5fC pairings do.

### MutSα complex recognizes G·5caC pairs in DNA substrates

**Figure 3:**
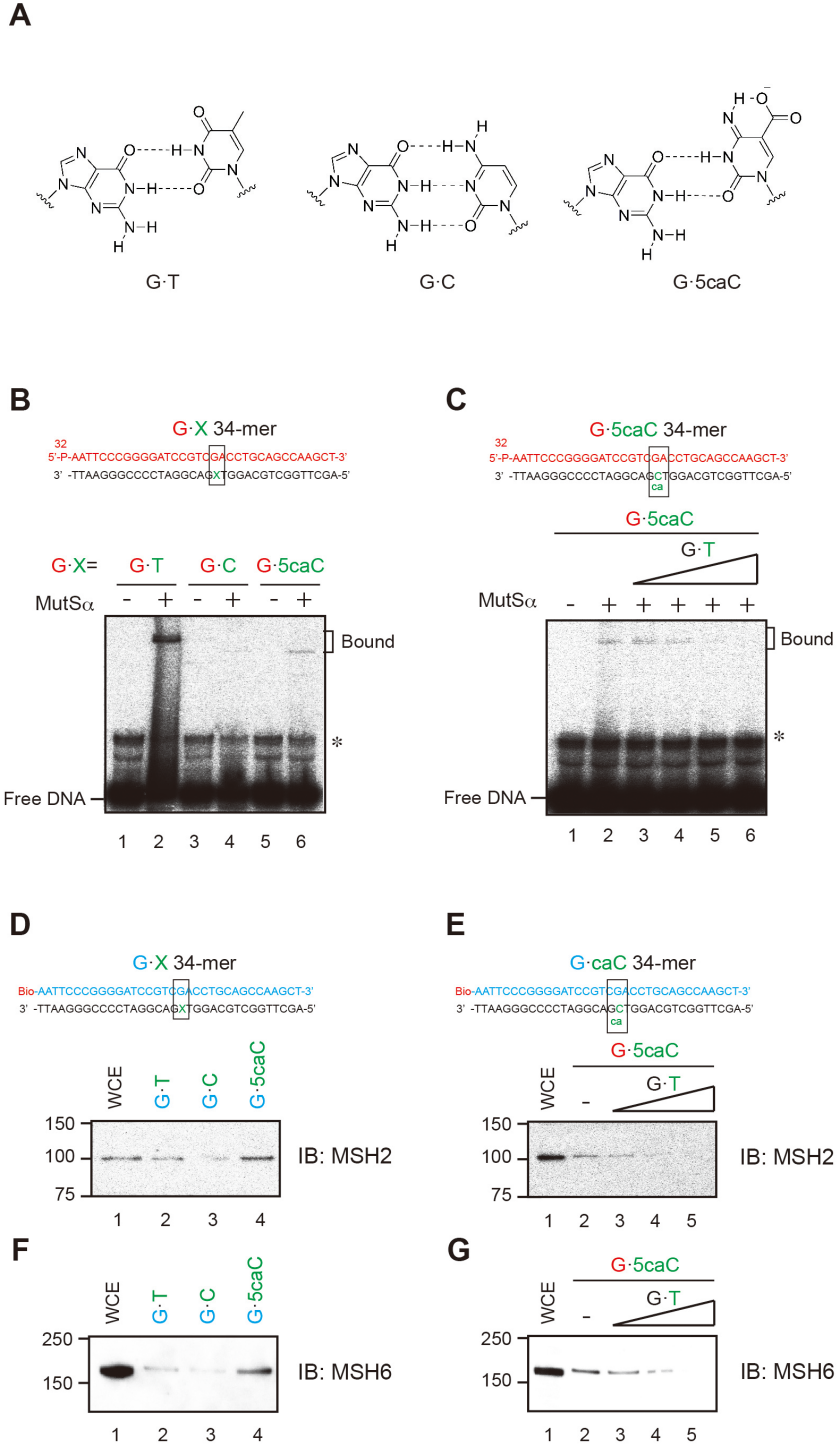
MutSα complex binds to G·5caC base pairs. (A) Postulated base-pairing models of 5caC with G. (B and C) A 34-mer oligonucleotide was 5′-labeled with ^32^P and annealed with a 34-mer oligonucleotide containing the 5caC. The mismatch substrates were incubated (B) with MutSα on ice for 20 min or (C) with MutSα and cold G·T mismatch substrates (lanes 3–6: non-labeled substrates/5′-labeled substrates molar ratio; ×1, ×10, ×50, and ×100) at 25°C for 20 min. Free and bound fractions were separated on nondenaturing 6% polyacrylamide gels containing 5 μM MgCl_2_. (D and E) Biotin-labeled 34-mer mismatch substrates were incubated (D) with whole cell extracts (10 μg) or (F) with whole cell extracts (10 μg) and G·T mismatch substrates (lanes 3–5: non-labeled substrates/5′-labeled substrates molar ratio; ×1, ×10, and ×50). Samples were loaded on 10% SDS-page gels, and MSH2 and MSH6 were detected by western blotting.

The proofreading function of DNA polymerases plays an important role in correcting replicative mismatch errors. Our results suggest this proofreading occurs at G·5caC pairings but not at other cytosine pairings. Although 5caC forms appropriate base pairs with guanine, we hypothesized that these pairings behave like mismatches (Figure 3A). If this holds true, the mismatch repair (MMR) protein MutS should recognize both pairings as it does G·T mismatches, which are a canonical MutS substrate^31^,^32^,^33^. To test this possibility, we performed electrophoretic mobility shift assays (EMSAs) with *Taq* MutS and 30-mer DNA substrates containing G·T, G·C, G·5mC, G·5hmC, G·5fC, and G·5caC. We observed a striking difference in MutS binding efficiency between these forms of cytosine. MutS bound G·T and G·5caC pairs (Figure S4A and S4B). The binding preference order was G·T = G·5caC > G·5fC > G·C = G·5mC = G·5hmC. Next, we performed EMSAs with 34-mer G·5caC-containing DNA substrates and human MMR protein MutSα complexes, which consist of MSH2 and MSH6 (Figure S5), because the exonuclease activity of Polδ was observed only on the 5caC templates. The MutSα complex is a human homolog of the MMR protein MutS and is indispensable for the mammalian MMR system^34^,^35^. MutSα bound to the positive control G·T pairs and to the G·5caC pairs (Figure 3B); addition of excess cold G·T DNA substrates inhibited binding between MutSα and G·5caC DNA substrates (Figure 3C). To confirm this interaction, biotin labeled-G·5caC DNA substrates were incubated with HeLa whole cell extracts and the DNA-bound proteins were pulled down with streptavidin-coated beads; MSH2 and MSH6 were detected by immunoblotting. Results confirmed the MutSα complex recognized G·5caC pairs in DNA substrates (Figures 3D and 3E). Thus, the G·5caC pairs behaved similarly to a G·T mismatch when Polδ synthesized new DNA fragments opposite 5caC, although DNA polymerase correctly incorporated dGTP. In addition, G·5caC pairs may be subjected to MMR in mammalian cells.

### Accumulation of 5caC affects cell proliferation

**Figure 4:**
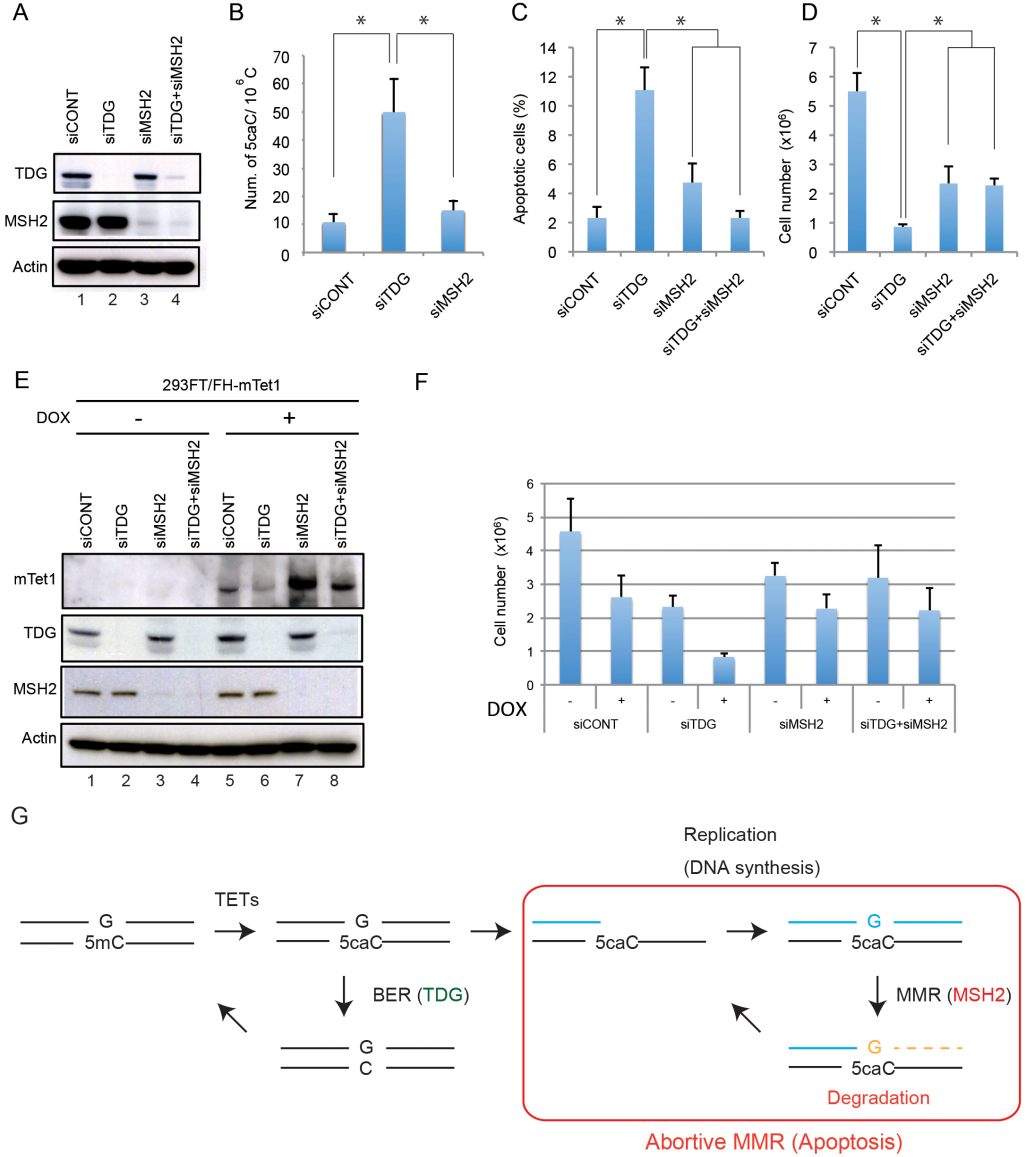
MSH2 knockdown rescued the cell death response in TDG-knockdown cells. (A) Western blotting of lysates prepared from siRNA-transfected HeLa cells using antibodies specific for the indicated proteins. Actin served as a loading control. (B) Mass spectrometric quantification of 5caC in siRNA-transfected HeLa cells. Data represent the average of at least three independent experiments and statistically analyzed by Student's t test (*p < 0.05). Error bars represent standard error of the mean. Three days after siRNA transfection, the percentage of apoptotic cells (C) and the number of viable cells (D) were calculated. Experiments were repeated six times and statistically analyzed by Student's t test (N = 6, *p < 0.01). Error bars represent standard deviation (SD). (E and F) 293 cells expressing mouse Tet1 under the control of Doxycycline were transfected with siRNA in the absence or presence of Doxycycline. Three days after siRNA transfection, expression of indicated proteins was examined by specific antibodies (E) and the number of viable cells was calculated (F). Experiments were repeated three times. Error bars represent SD. (G) Model for DNA damage effects of 5caC in DNA replication via abortive mismatch repair.

In the MMR system, exonuclease I removed the daughter DNA strand but could not remove template DNA containing the modified cytosine. Thus, the “offending” site persisted in the template. The ensuing abortive turnover of new DNA may result in a death response. Earlier studies have shown that TDG binds to these G·5caC pairs^36^ and excises the modified cytosine^20^. To investigate the effects of these G·T mismatch-mimicking base pairs in mammalian cells in vivo, we confirmed the expression levels of Tets, TDG, and MSH2 in various human cells (Figure S6) and then knocked down TDG expression, thereby inducing the accumulation of G·5caC base pairs and observed the viable cells in TDG-knockdown cultures (Figure 4A and Figure S7). As expected, 5caC was induced in TDG-knockdown cells (Figure 4B and Figure S8) versus control-knockdown or MSH2-knockdown cells. TDG-knockdown HeLa cells exhibited elevated apoptotic cell population and decreased number of surviving cells (Figure 4C and 4D), indicating the accumulated G·5caC base pairs are recognized by MMR, which induces the effects of DNA damage. This phenotype was partially rescued by knockdown of MSH2 expression. Once again, because Tet1-overexpressed 293 cells exhibit increased 5hmC and 5caC levels^19^, we investigated the effects of TDG knockdown in Tet1-overexpressed 293 cells (Figure 4E and Figure S9). When Tet1 expression was induced by treatment with doxycycline (Dox), cell number was reduced in all cases, indicating that Tet1-modifying cytosines behave as DNA lesions (Figure 4F). TDG knockdown leads to reduced cell numbers that were rescued by TDG-MSH2 double knockdown (Figure 4F). Thus, MMR was required for these DNA damage effects, which result in cell proliferation defects and decreased cell number.

## Discussion

In this study, we investigated the effects of oxidative forms of 5mC on DNA synthesis by replicative or translesion DNA polymerases with DNA templates containing a site-specific C, 5mC, 5hmC, 5fC, or 5caC. Although DNA polymerase correctly incorporated dGTP opposite any modified cytosine, DNA degradation products generated by the exonuclease activity of Polδ was significantly higher with 5caC than with other modified cytosines. Base pairing of guanine and the imino tautomer of 5caC (Figure 3A) has the same geometry as a G·T mismatch^37^ and was suggested in a previous study^38^; the results of exonucleolytic degradation in our study may be attributed to this type of base-pair formation. Münzel et al. demonstrated intramolecular hydrogen bonding between the amino and formyl groups of 5fC, but suggested that a substantial shift of tautomer equilibrium toward the imino form was unlikely^27^. Although it is very difficult to experimentally detect the unfavored imino tautomers of cytosine derivatives^39^,^40^, electron-withdrawing substituents at the C5 position of cytosine may facilitate base pair formation with the minor tautomer because this type of substitution destabilizes the Watson–Crick G·C base pair^41^. Formation of the G·5caC base pair in the same geometry as that of a G·T mismatch would stimulate the exonuclease activity of human Polδ.

In order to characterize the base-pair formation of the oxidized 5mC from another viewpoint, we examined binding of *Taq* MutS and human MutSα to C5-modified duplexes. As shown in Figures 3B and S4A, these proteins bound to the duplex containing 5caC; binding competition with a G·T mismatch-containing duplex was apparent (Figure 3C). MutS wedges a Phe side chain into the mismatch site, where this side chain is stacked onto one of the mismatched bases. This interaction changes the orientation of the stacked base, which originally formed the G·T-type mismatch shown in Figure 3A, so that a hydrogen bond forms between this base and the adjacent Glu. Then, bifurcated hydrogen bonds form between the thymine O4 and the nitrogen atoms of guanine in the MutS–DNA complex^31^,^32^. Our results support the formation of the G·5caC base pair shown in Figure 3A.

In living cells, DNA synthesis by DNA polymerase on 5caC, which cannot induce a typical mutation and cannot block DNA polymerase activity through replication, may also lead to adverse effects. Although TDG can remove these modified forms of cytosine from the genome, substantial amounts of 5caC remain in tissues and cells^19^. Therefore, when the 5caC on a DNA template pairs with the incoming dGTP via replicative DNA polymerase in the S phase, the delay in DNA synthesis may slow replication around the site and delay proper cell cycle progression. We showed that DNA polymerase generated G·T mismatch-mimicking G·5caC pairs that were recognized by the MutSα complex. MMR can eliminate the G·T mismatch-mimicking base pairs to remove the daughter strand, but cannot remove the modified cytosine. This process may induce abortive turnover of DNA synthesis (Figure 4G). As shown in Figure 4, MSH2 knockdown rescued the cell death phenotype induced by TDG knockdown. We suggest the G·T mismatch-mimicking base pairs formed by 5caC behaved as DNA lesions processed by MMR. This scenario is similar to a model of apoptosis triggered by O^6^-methylguanine (O^6^-meG)^34^,^35^, which gives rise to O^6^-meG·T mismatches that are subject to abortive MMR and apoptosis. Therefore, 5caC induced by knockdown of TDG may drive genomic instability in mammalian cells, similar to O^6^-meG. TDG knockout leads to embryonic lethality in mice, presumably due to aberrant epigenetic modifications, especially DNA methylation status and transcriptional defects^21^,^22^. However, G·T mismatch-mimicking G·5caC pairs may also contribute to embryonic lethality in TDG-knockout mice. Additionally, because 5caC residues behave as DNA lesions that slow DNA replication via the exonuclease function of DNA polymerase, it may be removed before replication to prevent formation of lethal DNA lesions.

MSH2 knockdown partially rescued the TDG-knockdown phenotype. This suggests that other effects of 5caC persist, independent of replication and MMR. Because the 5caC on transcribed strands induces transcriptional pausing^42^, a part of the residual lethality may be attributed to lesions encountered during transcription. Spruijt et al. reported that 5caC recruits a large number of DNA repair proteins in mouse ES cells, including BER (Neil1, Neil3, and Mpg) and MMR (Msh3 and Exo1)^43^. Thus, these DNA repair proteins may be involved in removing 5caC from genomic DNA to rescue the TDG-knockdown phenotype under certain circumstances. Interestingly, in TDG-knockdown mouse ES cells, no apoptotic effects are observed^23^. This may support the observation that 5caC decarboxylation exists in mouse ES but not HeLa cells^44^. This activity might determine the extent of cell viability in cells with accumulated 5caC DNA lesions.

Our results indicated that the electrostatic repulsion of oxidatively modified cytosines 5caC paired with guanine influenced the exonuclease activity of DNA polymerases and the damage recognition step of MMR. This process may lead to abortive turnover of MMR. Thus, 5caC residues that were assumed to be intermediates for an active demethylation pathway may be oxidative DNA lesions that must be removed before replication. These findings provide an important new perspective on the potential functional interplay between cytosine modification status and replication.

## Methods

### DNA substrates

Thirty-mer DNA substrates containing 5mC or 5hmC were synthesized at Tsukuba Oligo Service and purified by high-performance liquid chromatography (HPLC). DNA substrates containing 5fC or 5caC were synthesized in an Applied Biosystems 3400 DNA synthesizer (Applied Biosystems) by using phosphoramidite building blocks purchased from Glen Research and were purified by HPLC. The oligonucleotides were 5′-phosphorylated using (γ-^32^P)-ATP (PerkinElmer Life Sciences) and T4 phosphoramidite kinase (TaKaRa). Unincorporated nucleotides were removed using MicroSpin G-25 columns (GE Healthcare).

### In vitro DNA synthesis assays

DNA synthesis assays were performed as described^30^. Briefly, the 5′-^32^P-labeled primer-template complex was prepared by mixing the primer with a template containing the indicated sequence context at a molar ratio of 1:1. Ten-microliter reaction mixtures containing 10 mM Tris-HCl (pH 7.9), 50 mM NaCl, 10 mM MgCl_2_, 40 nM of a labeled primer-template complex, and the indicated DNA polymerases were incubated. The reactions were terminated by adding 10 μL of stop solution containing 95% formamide, 10 mM EDTA, 0.025% bromophenol, and 0.025% xylene cyanol. The fragments were separated by electrophoresis on a denaturing polyacrylamide gel, dried, and analyzed using a Fuji FLA-7000 phosphorimager (Fujifilm).

### EMSAs

Standard binding reaction mixtures (10 μL) contained 1 nM ^32^P-labeled substrate and the purified 2 nM MutSα complex in binding buffer A containing 10 mM HEPES-KOH (pH 7.7), 50 mM KCl, 2 mM MgCl_2_, 1 mM EDTA, 1 mM DTT, and 0.1 mM ADP. The reaction mixtures were incubated, and loading buffer (2 μL) containing 50% glycerol, 0.5% bromophenol blue, and 0.5% xylene cyanol was added^45^. The samples were separated on nondenaturing 6% polyacrylamide gels.

### MutSα complex binding assays

Binding reaction mixtures (20 μL) contained 1 μM biotin-labeled 34-mer mismatch substrate, poly dI-dC (1 μg), and HeLa cell extracts (10 μg) in binding buffer A. The reaction mixtures were incubated at 4°C for 60 min and then with 5 μL DynaBeads M-280 (Invitrogen) for 30 min. Unbound proteins were removed by washing the beads three times with binding buffer A containing 1% Triton X-100. Bound fractions were separated on 10% SDS-page gels and analyzed by western blotting with 0.25 ng MSH2 antibodies (G219-1129, BD Biosciences) or 0.25 ng MSH6 antibodies (44/MSH6, BD Biosciences).

### Knockdown experiments

siRNA duplexes specifically targeting TDG (SI02665040) and MSH2 (SI02663563) and nontargeting control siRNAs (1027280) were purchased from Qiagen and transfected into cells using Lipofectamine RNAiMAX (Invitrogen) according to the manufacturer's instructions. Four days after siRNA transfection, the cells were trypsinized and viable cells were counted. Total RNA was isolated using RNeasy Mini Kit (Qiagen) and cDNA was generated with the SuperScript VILO Master Mix (Invitrogen). Real-time quantitative PCR (qPCR) was performed on an Mx3005P QPCR System (Agilent Technologies) using SYBR Green reagent (Roche Applied Science). cDNA levels of the target genes were analyzed by the comparative Ct method and normalized to ACTB. qPCR primers are listed in the Supplementary Table. To quantify apoptotic cells, a Tali Apoptosis Kit - Annexin V Alexa Fluor 488 and Propidium Iodide (Invitrogen) was used according to the manufacturer's instructions. Mass spectrometry analyses to quantify 5caC were performed as previously described^19^.

## Author Contributions

S.Ito. and I.K. conceived the project; T.S., S.Ito., M.T., R.K., L.C., M.S., M.U. and I.K. conducted experiments; S.Ito., H.K., Y.M., J.S., C.M., F.H., S.I. and I.K. interpreted the data; S.Ito., S.I. and I.K. wrote the manuscript. All authors discussed the results and commented on the manuscript.

## Supplementary Material

Supplementary Information Supplementary information

## References

[b1] Reik W. Stability and flexibility of epigenetic gene regulation in mammalian development. Nature 447, 425–432 (2007). 17522676 10.1038/nature05918

[b2] Goll M. G. & Bestor T. H. Eukaryotic cytosine methyltransferases. Annu. Rev. Biochem. 74, 481–514 (2005). 15952895 10.1146/annurev.biochem.74.010904.153721

[b3] Ito S. *et al.* Role of Tet proteins in 5mC to 5hmC conversion, ES-cell self-renewal and inner cell mass specification. Nature 466, 1129–1133 (2010). 20639862 10.1038/nature09303 PMC3491567

[b4] Tahiliani M. *et al.* Conversion of 5-methylcytosine to 5-hydroxymethylcytosine in mammalian DNA by MLL partner TET1. Science 324, 930–935 (2009). 19372391 10.1126/science.1170116 PMC2715015

[b5] Kriaucionis S. & Heintz N. The Nuclear DNA Base 5-Hydroxymethylcytosine Is Present in Purkinje Neurons and the Brain. Science 324, 929–930 (2009). 19372393 10.1126/science.1169786 PMC3263819

[b6] Wu H. *et al.* Genome-wide analysis of 5-hydroxymethylcytosine distribution reveals its dual function in transcriptional regulation in mouse embryonic stem cells. Genes Dev. 25, 679–684 (2011). 21460036 10.1101/gad.2036011 PMC3070931

[b7] Williams K. *et al.* TET1 and hydroxymethylcytosine in transcription and DNA methylation fidelity. Nature 473, 343–348 (2011). 21490601 10.1038/nature10066 PMC3408592

[b8] Ficz G. *et al.* Dynamic regulation of 5-hydroxymethylcytosine in mouse ES cells and during differentiation. Nature 473, 398–402 (2011). 21460836 10.1038/nature10008

[b9] Song C.-X. *et al.* Selective chemical labeling reveals the genome-wide distribution of 5-hydroxymethylcytosine. Nat. Biotechnol. 29, 68–72 (2011). 21151123 10.1038/nbt.1732 PMC3107705

[b10] Pastor W. A. *et al.* Genome-wide mapping of 5-hydroxymethylcytosine in embryonic stem cells. Nature 473, 394–397 (2011). 21552279 10.1038/nature10102 PMC3124347

[b11] Booth M. J. *et al.* Quantitative Sequencing of 5-Methylcytosine and 5-Hydroxymethylcytosine at Single-Base Resolution. Science 336, 934–937 (2012). 22539555 10.1126/science.1220671

[b12] Yu M. *et al.* Base-resolution analysis of 5-hydroxymethylcytosine in the mammalian genome. Cell 149, 1368–1380 (2012). 22608086 10.1016/j.cell.2012.04.027 PMC3589129

[b13] Xu Y. *et al.* Genome-wide regulation of 5hmC, 5mC, and gene expression by Tet1 hydroxylase in mouse embryonic stem cells. Mol. Cell 42, 451–464 (2011). 21514197 10.1016/j.molcel.2011.04.005 PMC3099128

[b14] Globisch D. *et al.* Tissue distribution of 5-hydroxymethylcytosine and search for active demethylation intermediates. PLoS ONE 5, e15367 (2010). 21203455 10.1371/journal.pone.0015367 PMC3009720

[b15] Szwagierczak A., Bultmann S., Schmidt C. S., Spada F. & Leonhardt H. Sensitive enzymatic quantification of 5-hydroxymethylcytosine in genomic DNA. Nucleic Acids Res. 38, e181 (2010). 20685817 10.1093/nar/gkq684 PMC2965258

[b16] Hashimoto H. *et al.* Recognition and potential mechanisms for replication and erasure of cytosine hydroxymethylation. Nucleic Acids Res. 40, 4841–4849 (2012). 22362737 10.1093/nar/gks155 PMC3367191

[b17] Valinluck V. & Sowers L. C. Endogenous cytosine damage products alter the site selectivity of human DNA maintenance methyltransferase DNMT1. Cancer Res. 67, 946–950 (2007). 17283125 10.1158/0008-5472.CAN-06-3123

[b18] He Y.-F. *et al.* Tet-mediated formation of 5-carboxylcytosine and its excision by TDG in mammalian DNA. Science 333, 1303–1307 (2011). 21817016 10.1126/science.1210944 PMC3462231

[b19] Ito S. *et al.* Tet proteins can convert 5-methylcytosine to 5-formylcytosine and 5-carboxylcytosine. Science 333, 1300–1303 (2011). 21778364 10.1126/science.1210597 PMC3495246

[b20] Maiti A. & Drohat A. C. Thymine DNA glycosylase can rapidly excise 5-formylcytosine and 5-carboxylcytosine: potential implications for active demethylation of CpG sites. J. Biol. Chem. 286, 35334–35338 (2011). 21862836 10.1074/jbc.C111.284620 PMC3195571

[b21] Cortázar D. *et al.* Embryonic lethal phenotype reveals a function of TDG in maintaining epigenetic stability. Nature 470, 419–423 (2011). 21278727 10.1038/nature09672

[b22] Cortellino S. *et al.* Thymine DNA Glycosylase Is Essential for Active DNA Demethylation by Linked Deamination-Base Excision Repair. Cell 146, 67–79 (2011). 21722948 10.1016/j.cell.2011.06.020 PMC3230223

[b23] Shen L. *et al.* Genome-wide Analysis Reveals TET- and TDG-Dependent 5-Methylcytosine Oxidation Dynamics. Cell 153, 692–706 (2013). 23602152 10.1016/j.cell.2013.04.002 PMC3687516

[b24] Song C.-X. *et al.* Genome-wide Profiling of 5-Formylcytosine Reveals Its Roles in Epigenetic Priming. Cell 153, 678–691 (2013). 23602153 10.1016/j.cell.2013.04.001 PMC3657391

[b25] Kamiya H. *et al.* Mutagenicity of 5-formylcytosine, an oxidation product of 5-methylcytosine, in DNA in mammalian cells. J. Biochem. 132, 551–555 (2002). 12359069 10.1093/oxfordjournals.jbchem.a003256

[b26] Karino N., Ueno Y. & Matsuda A. Synthesis and properties of oligonucleotides containing 5-formyl-2′-deoxycytidine: in vitro DNA polymerase reactions on DNA templates containing 5-formyl-2′-deoxycytidine. Nucleic Acids Res. 29, 2456–2463 (2001). 11410651 10.1093/nar/29.12.2456 PMC55734

[b27] Münzel M. *et al.* Improved synthesis and mutagenicity of oligonucleotides containing 5-hydroxymethylcytosine, 5-formylcytosine and 5-carboxylcytosine. Chemistry 17, 13782–13788 (2011). 22069110 10.1002/chem.201102782

[b28] Xing X.-W. *et al.* Mutagenic and cytotoxic properties of oxidation products of 5-methylcytosine revealed by next-generation sequencing. PLoS ONE 8, e72993 (2013). 24066027 10.1371/journal.pone.0072993 PMC3774748

[b29] Johnson R. E., Kondratick C. M., Prakash S. & Prakash L. hRAD30 mutations in the variant form of xeroderma pigmentosum. Science 285, 263–265 (1999). 10398605 10.1126/science.285.5425.263

[b30] Masutani C. *et al.* The XPV (xeroderma pigmentosum variant) gene encodes human DNA polymerase eta. Nature 399, 700–704 (1999). 10385124 10.1038/21447

[b31] Lamers M. H. *et al.* The crystal structure of DNA mismatch repair protein MutS binding to a GT mismatch. Nature 407, 711–717 (2000). 11048711 10.1038/35037523

[b32] Natrajan G. *et al.* Structures of Escherichia coli DNA mismatch repair enzyme MutS in complex with different mismatches: a common recognition mode for diverse substrates. Nucleic Acids Res. 31, 4814–4821 (2003). 12907723 10.1093/nar/gkg677 PMC169951

[b33] Obmolova G., Ban C., Hsieh P. & Yang W. Crystal structures of mismatch repair protein MutS and its complex with a substrate DNA. Nature 407, 703–710 (2000). 11048710 10.1038/35037509

[b34] Branch P., Hampson R. & Karran P. DNA mismatch binding defects, DNA damage tolerance, and mutator phenotypes in human colorectal carcinoma cell lines. Cancer Res. 55, 2304–2309 (1995). 7757980

[b35] Karran P. & Bignami M. Drug-related killings: a case of mistaken identity. Chem. Biol. 3, 875–879 (1996). 8939713 10.1016/s1074-5521(96)90175-1

[b36] Zhang L. *et al.* Thymine DNA glycosylase specifically recognizes 5-carboxylcytosine-modified DNA. Nat. Chem. Biol. 8, 328–330 (2012). 22327402 10.1038/nchembio.914 PMC3307914

[b37] Allawi H. T. & SantaLucia J. NMR solution structure of a DNA dodecamer containing single G·T mismatches. Nucleic Acids Res. 26, 4925–2934 (1998). 9776755 10.1093/nar/26.21.4925 PMC147937

[b38] Hashimoto H., Hong S., Bhagwat A. S., Zhang X. & Cheng X. Excision of 5-hydroxymethyluracil and 5-carboxylcytosine by the thymine DNA glycosylase domain: its structural basis and implications for active DNA demethylation. Nucleic Acids Res. 40, 10203–10214 (2012). 22962365 10.1093/nar/gks845 PMC3488261

[b39] La Francois C. J., Jang Y. H., Cagin T., Goddard W. A. & Sowers L. C. Conformation and proton configuration of pyrimidine deoxynucleoside oxidation damage products in water. Chem. Res. Toxicol. 13, 462–470 (2000). 10858319 10.1021/tx990209u

[b40] Suen W., Spiro T. G., Sowers L. C. & Fresco J. R. Identification by UV resonance Raman spectroscopy of an imino tautomer of 5-hydroxy-2′-deoxycytidine, a powerful base analog transition mutagen with a much higher unfavored tautomer frequency than that of the natural residue 2′-deoxycytidine. Proc. Natl. Acad. Sci. U.S.A. 96, 4500–4505 (1999). 10200291 10.1073/pnas.96.8.4500 PMC16361

[b41] Xue C. & Popelier P. L. A. Prediction of Interaction Energies of Substituted Hydrogen-Bonded Watson−Crick Cytosine:Guanine 8XBase Pairs. J. Phys. Chem. B 113, 3245–3250 (2009). 19260717 10.1021/jp8071926

[b42] Kellinger M. W. *et al.* 5-formylcytosine and 5-carboxylcytosine reduce the rate and substrate specificity of RNA polymerase II transcription. Nat. Struct. Mol. Biol. 19, 831–833 (2012). 22820989 10.1038/nsmb.2346 PMC3414690

[b43] Spruijt C. G. *et al.* Dynamic readers for 5-(hydroxy)methylcytosine and its oxidized derivatives. Cell 152, 1146–1159 (2013). 23434322 10.1016/j.cell.2013.02.004

[b44] Schiesser S. *et al.* Mechanism and stem-cell activity of 5-carboxycytosine decarboxylation determined by isotope tracing. Angew. Chem. Int. Ed. Engl. 51, 6516–6520 (2012). 22644704 10.1002/anie.201202583

[b45] Hsieh P. Identification and Characterization of a Thermostable MutS Homolog from Thermus aquaticus. Journal of Biological Chemistry 271, 5040–5048 (1996). 8617781 10.1074/jbc.271.9.5040

