# Supplementary material for: Guanine- 5-carboxylcytosine base pairs mimic mismatches during DNA replication
Source: Sci Rep. 2014 Jun 9;4:5220. doi: 10.1038/srep05220 (PMC4048885; doi:10.1038/srep05220)
Supplement: Supplementary Information [file srep05220-s1.doc]

**Supplementary Information**

**Guanine- 5-carboxylcytosine base pairs mimic mismatches during DNA replication**

**Toshihiro Shibutani^1^, Shinsuke Ito^2^*, Mariko Toda^1^, Rie Kanao^3^, Leonard B. Collins^4^, Marika Shibata^2^, Miho Urabe^2^, Haruhiko Koseki^2^, Yuji Masuda^3,5^, James A. Swenberg^4^, Chikahide Masutani^3^, Fumio Hanaoka^6^, Shigenori Iwai^1^, Isao Kuraoka^1^***

^1^Graduate School of Engineering Science, Osaka University Graduate School of Engineering Science, 1-3 Machikaneyama, Toyonaka, Osaka 560-8531 Japan; ^2^Laboratory for Developmental Genetics, RIKEN Research Center for Allergy and Immunology, Yokohama 230-0045, Japan; ^3^Research Institute of Environmental Medicine, Nagoya University, Furo-cho, Chikusa-ku, Nagoya 464-8601, Japan; ^4^Curriculum in Toxicology and Department of Environmental Sciences and Engineering, ^5^Department of Environmental Sciences and Engineering, Gillings School of Global Public Health, University of North Carolina at Chapel Hill Chapel Hill, NC 27599, USA. ^6^Faculty of Science, Gakushuin University, 1-5-1 Mejiro, Toshima-ku, Tokyo 171-8588, Japan.

*Corresponding authors: Shinsuke Ito, Laboratory for Developmental Genetics, RIKEN Research Center for Allergy and Immunology, Yokohama 230-0045, E-mail: [shinsuke_ito@rcai.riken.jp](mailto:shinsuke_ito@rcai.riken.jp) ; Isao Kuraoka, Graduate School of Engineering Science, Osaka University, 1-3 Machikaneyama, Toyonaka, Osaka 560-8531, Japan. Tel: +81-6-6850-6250, Fax: +81-6-6850-6240, E-mail: [Kuraoka@chem.es.osaka-u.ac.jp](mailto:Kuraoka@chem.es.osaka-u.ac.jp)

**
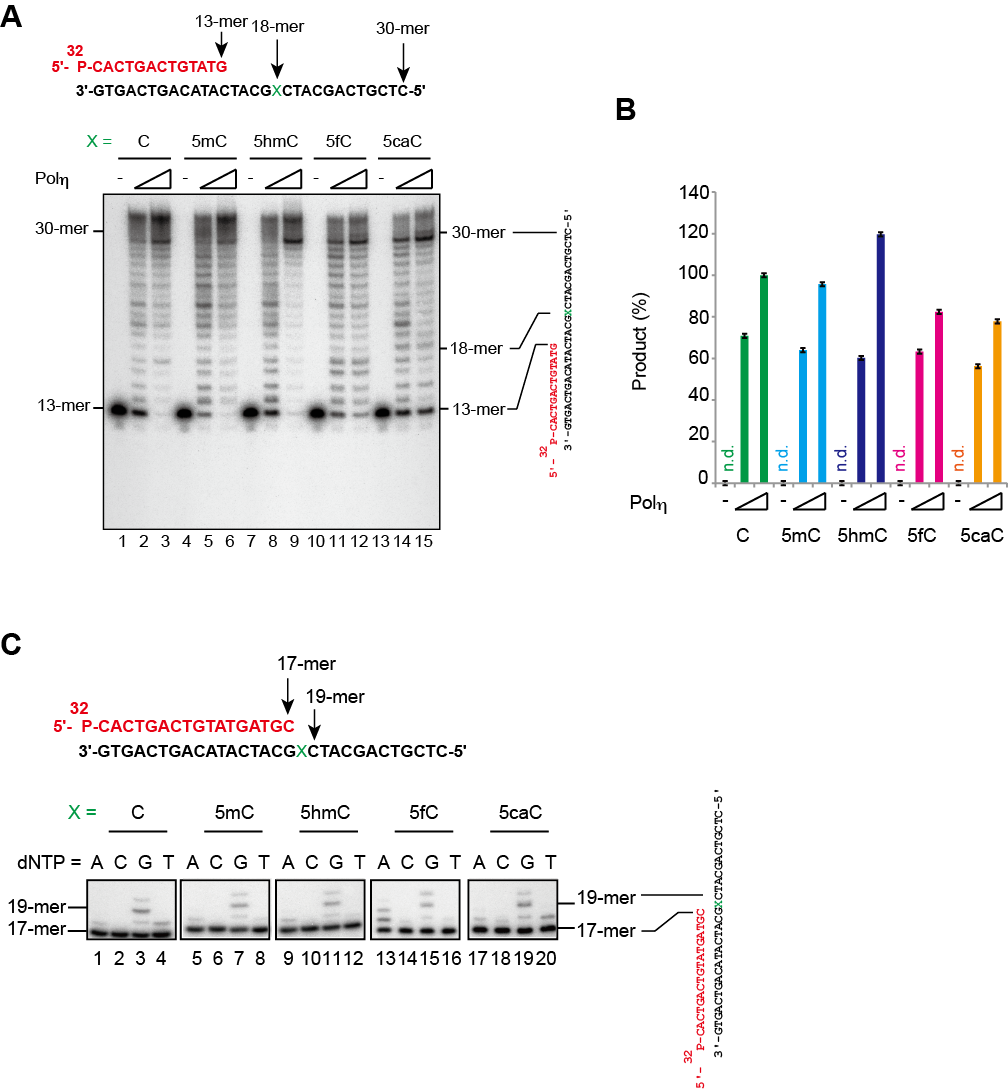
**

Figure S1. **DNA synthesis reactions of human Polη on 5caC DNA templates.** (A and B) A 13-mer primer was 5′-labeled with ^32^P and annealed with a 30-mer oligonucleotide containing the indicated modified cytosines at position X (upper panel). The primer/template complexes were incubated (A) with increasing amounts of Polη (0, 5, and 10 ng in each group of 3 lanes) at 30°C for 5 min. (B) Relative DNA synthesis efficiency on all 5 modified cytosine templates. Data were normalized to DNA synthesis efficiency of Polη (10 ng) for normal cytosine-containing templates (lane 3). Quantification of the 30–31 nt fragments by image analysis. The error bars indicate the standard deviation. n.d.: not determined. (C) A 17-mer primer was 5′-labeled with ^32^P and annealed with a 30-mer oligonucleotide containing the indicated modified cytosines at position X (upper panel). The primer/template complexes were incubated with Polη (1 ng) at 30°C for 5 min with one of the indicated dNTPs (lanes 1-4, 5-8, 9-12, 13-16, and 17-20).


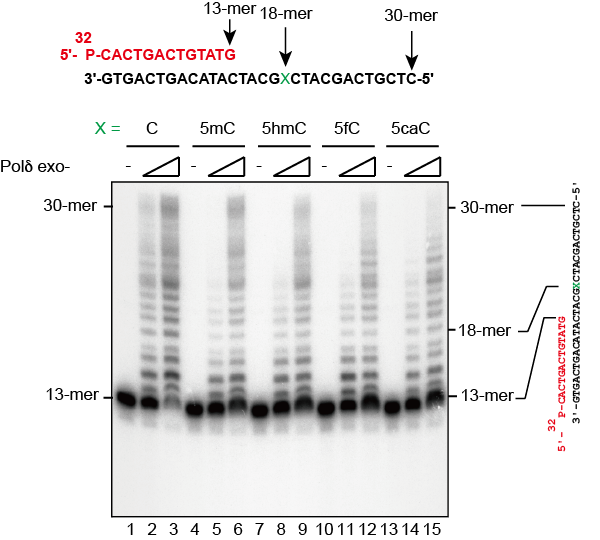


Figure S2. **DNA synthesis reactions of human Polδ exo- on 5caC DNA templates.** (A and B) A 13-mer primer was 5′-labeled with ^32^P and annealed with a 30-mer oligonucleotide containing the indicated modified cytosines at position X (upper panel). The primer/template complexes were incubated (A) with increasing amounts of Pold exo- (0, 22.5, and 45 ng in each group of 3 lanes) at 30°C for 5 min.


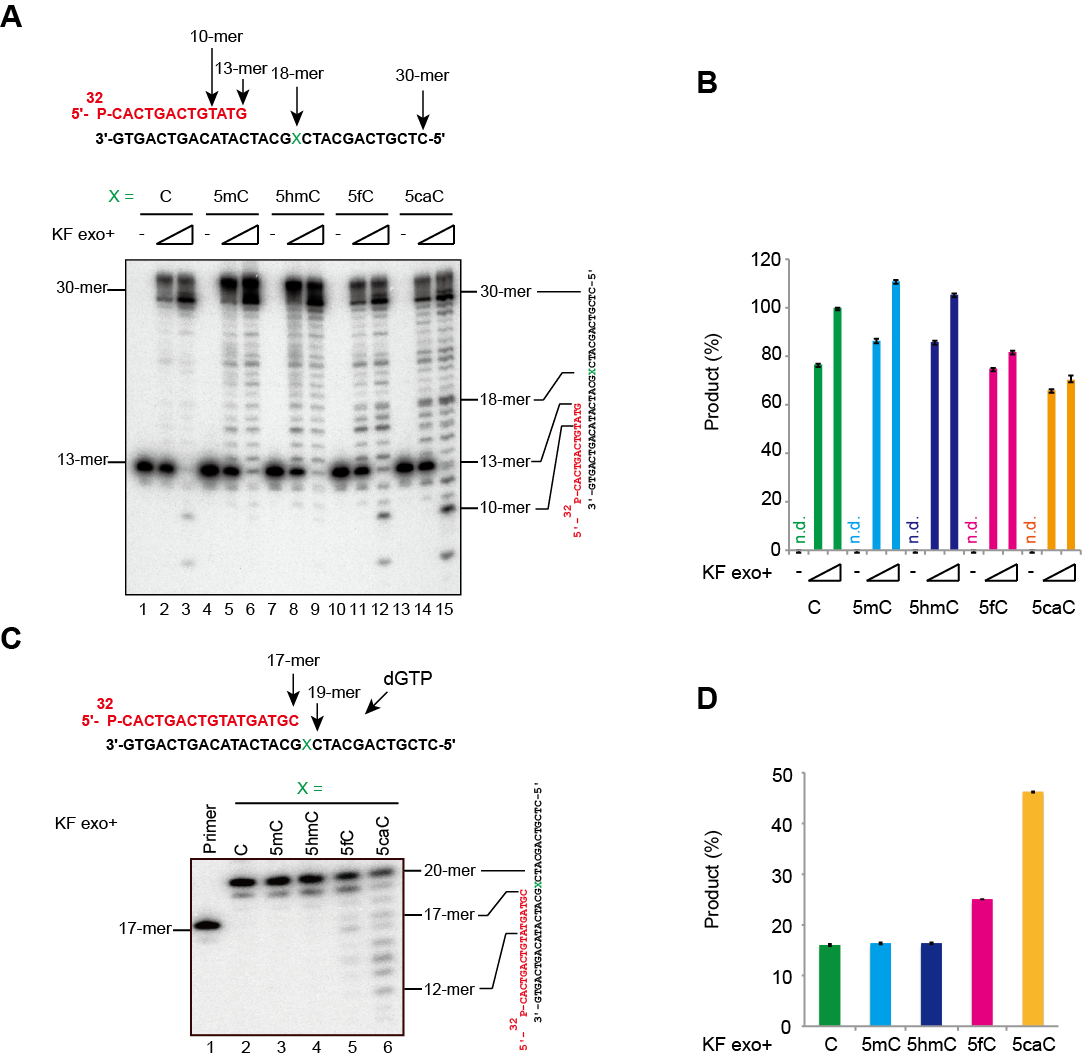


Figure S3. **DNA synthesis reactions of Klenow fragment (KF exo+) on DNA templates containing 5fC and 5caC.** (A) A 13-mer primer was 5′-labeled with ^32^P and annealed with a 30-mer oligonucleotide containing the indicated modified cytosines at position X (upper panel). The primer/template complexes were incubated with increasing amounts of KF exo+ (0, 0.04, and 1 U in each group of 3 lanes) at 30°C for 5 min. (B) Relative DNA synthesis efficiency on all 5 modified cytosine templates by KF exo+ . Data were normalized to DNA synthesis efficiency of KF exo+ (1 units) for normal cytosine-containing templates (lane 3). Quantification of the 30–31 nt fragments by image analysis. The error bars indicate the standard deviation. n.d.: not determined. (C) A 17-mer primer was 5′-labeled with ^32^P and annealed with a 30-mer oligonucleotide containing the indicated modified cytosines at position X (upper panel). The 5′-labeled 17-mer primer was loading in the first lane. The primer/template complexes were incubated with KF exo+ (1 U) and dGTP (100 µM) at 30°C for 5 min. (D) Relative exonuclease efficiency of DNA polymerase on all 5 modified cytosine templates by KF exo+. Data were normalized to exonuclease efficiency of KF exo+ for normal cytosine-containing templates (lane 1). Quantification of the degradation fragments by image analysis. The error bars indicate the standard deviation. n.d.: not determined.


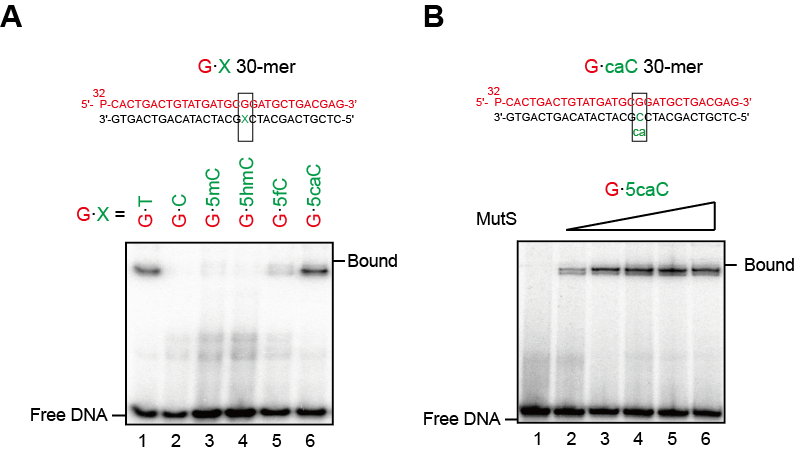


Figure S4. ***Taq* MutS binds to G·5caC base pairs.** (A) A 30-mer oligonucleotide was 5′-labeled with ^32^P and annealed with a 30-mer oligonucleotide containing the indicated modified cytosines. The mismatch substrates were incubated with *Taq* MutS (0.5 µg) at 65°C for 30 min. Free and bound fractions were separated on nondenaturing 8 % polyacrylamide gels. (B) The G·5caC mismatch substrates were incubated with increasing amounts of *Taq* MutS (0, 0.1, 0.2, 0.3, 0.4, or 0.5 µg) at 65°C for 30 min.


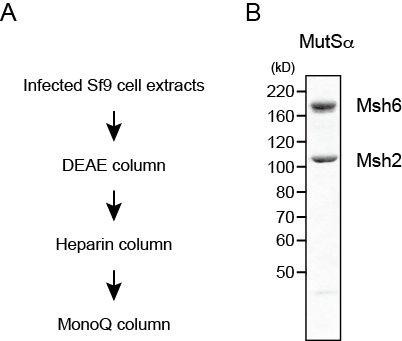


Figure S5. **Purification of MutSa complex.** (A) Experimental procedure for purification of the MutSa complex (Kanao et al., 2009). (B) Purified MutSa complex were loaded on 7.5% SDS-page gel and visualized by staining with Coomassie Brilliant Blue.


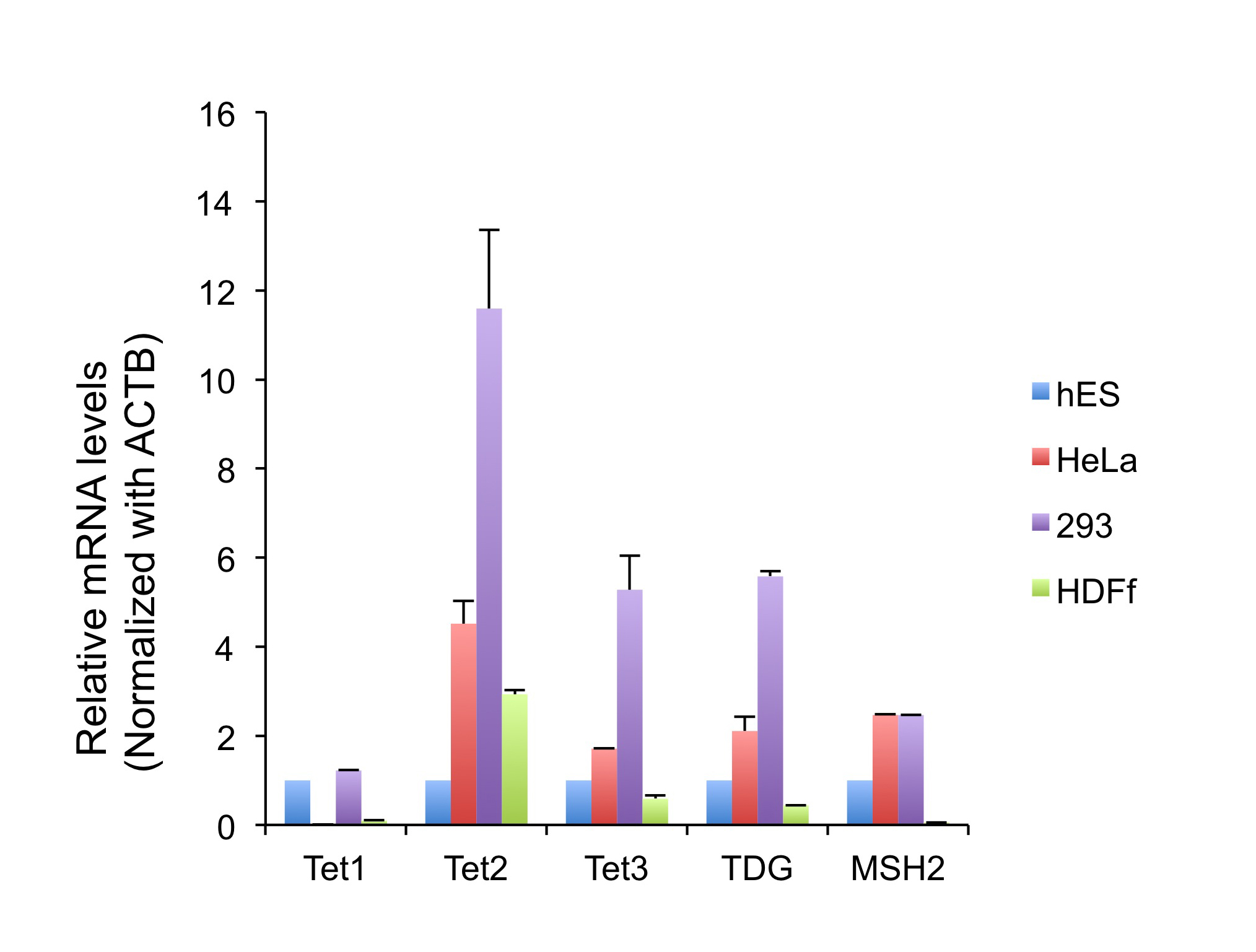


Figure S6. **Relative expression levels of Tets, TDG, and MSH2 in various human cells.** RT-qPCR analysis of expression levels of Tets, TDG, and MSH2 in hES, HeLa, 293, and HDFf (fetal Human Dermal Fibroblast) cells. The expression level in human ES cells is set as 1 for each of the genes. Data shown is the average with error bars (SD).


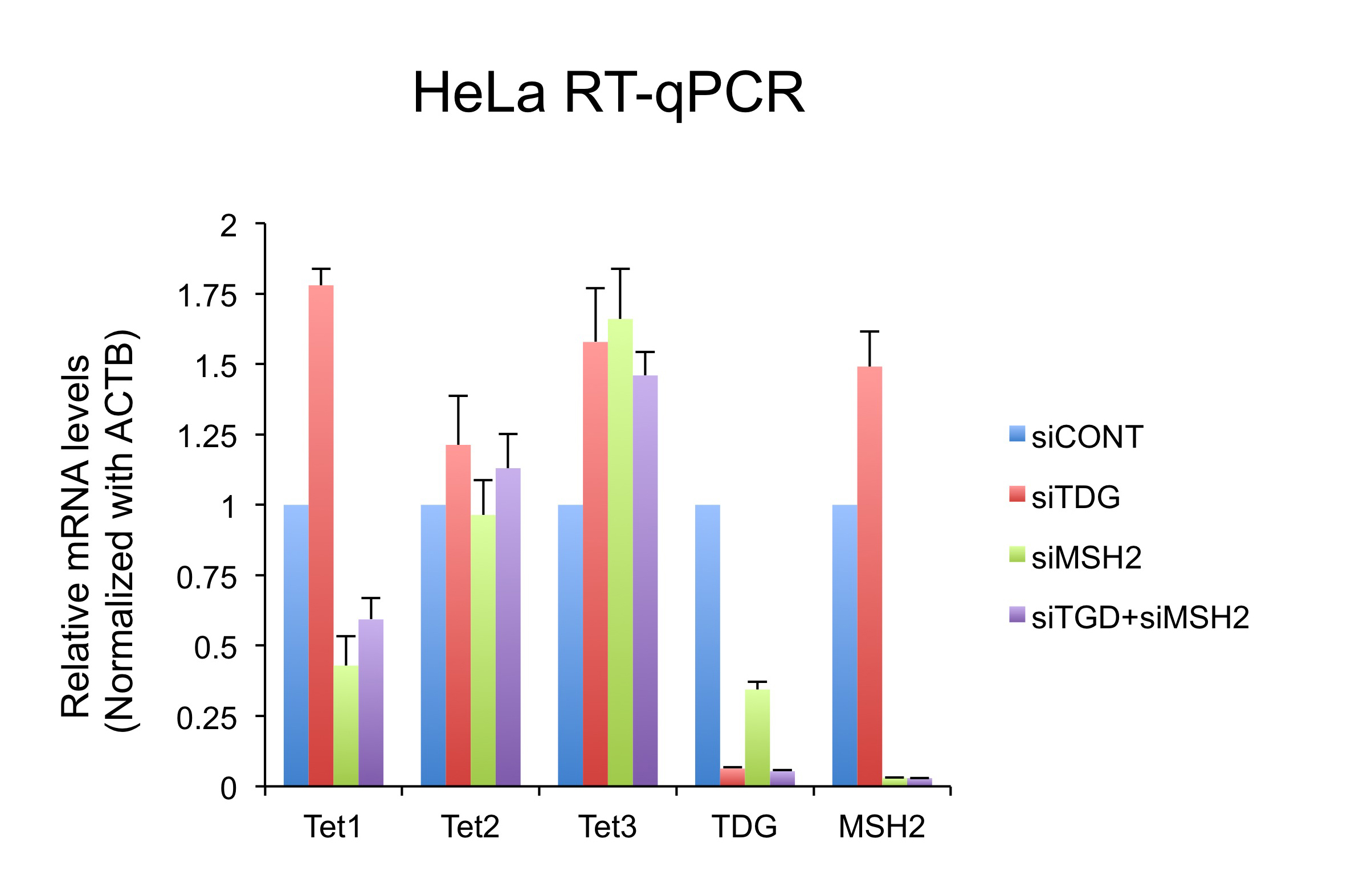


Figure S7. **Relative expression levels of Tets, TDG, and MSH2 in HeLa cells transfected with siRNAs.** RT-qPCR analysis of expression levels of Tets, TDG, and MSH2 in HeLa cells transfected with indicated siRNAs. The expression level in siCONT-transfected HeLa cells is set as 1 for each of the genes. Data shown is the average with SD.


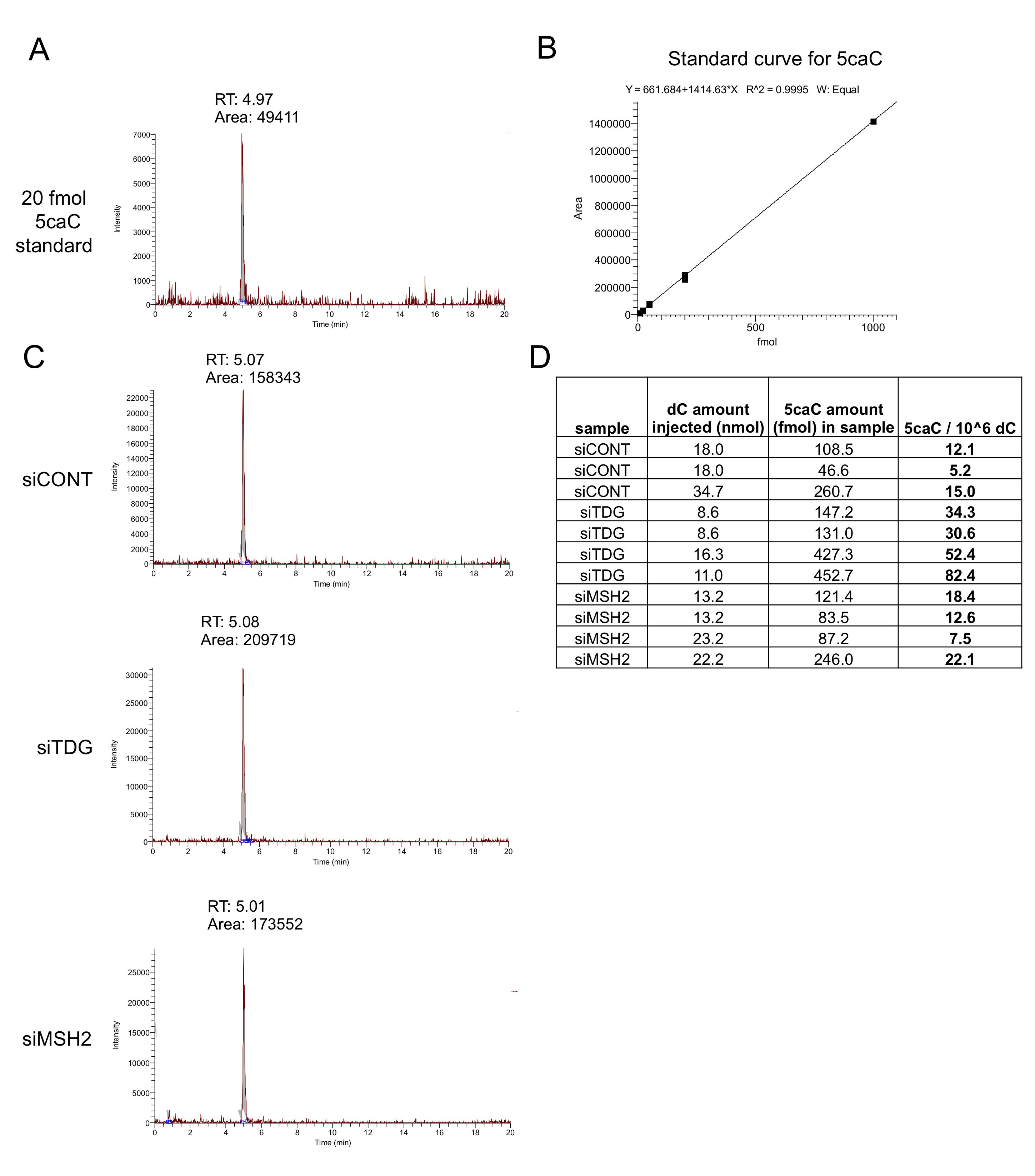


Figure S8. **Quantificaton of 5caC in genomic DNA of HeLa cells transfected with siRNAs.** (A) Detection of 5caC standard (20 fmol) loaded on the column by LC-MS/MS. (B) Standard curve for quantification of 5caC. 10, 20, 50, 200, and 1000 fmol of 5caC standard were used to prepare the standard curve. (C) Mass spectrometry detection of 5caC in genomic DNA of HeLa cells transfected with indicated siRNAs. Typical LC-MS/MS chromatograms are shown. (D) The amounts of 5caC were calculated with the standard curve (B).


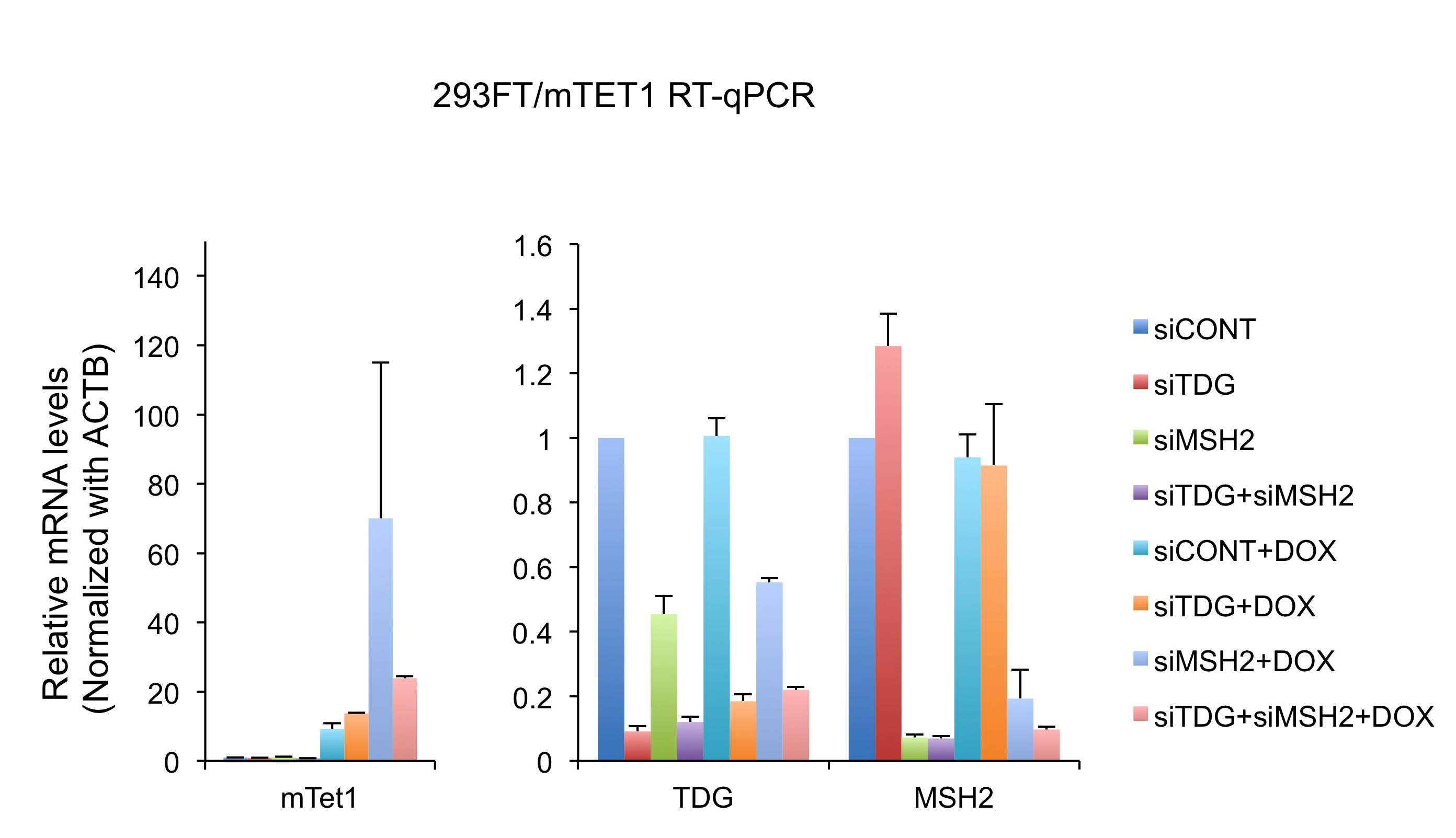


Figure S9. **Relative expression levels of mouse Tet1, TDG, and MSH2 in 293FT cells that express mouse Tet1 under the control of Doxycycline and are transfected with siRNAs.** RT-qPCR analysis of expression levels of mouse Tet1, TDG, and MSH2 in 293FT cells transfected with indicated siRNAs. Indicated cells were cultured in the presence of Doxycycline.The expression level in siCONT-transfected 293FT cells is set as 1 for each of the genes. Data shown is the average with SD.

Supplementary table.

RT-qPCR primer sequences

| gene Forward primer  Reverse primer |
| --- |

TDG 5’-TCACACTCTACCAGGGAAGTATG-3’

5’-ACGTCCTCCTTCACGAAATTCT-3’

MSH2 5’-CACTGTCTGCGGTAATCAAGT-3’

5’-CTCTGACTGCTGCAATATCCAAT-3’

ACTB 5’-TGGCACCCAGCACAATGAA-3’

5’-CTAAGTCATAGTCCGCCTAGAAGCA-3’

TET1 5’-AACAAGAGGCCCCAGAG-3

5’-TTCTTCCCCATGACCAC-3’

TET2 5’-AAACAAACTGAAAACGCAAG-3’

5’-GTGGTGGCTGCTTCTGTAG-3’

TET3 5’-AAGAGTCTGCTGGACACACC-3’

5’-CTCCATGAGTTCCCGGAATAG-3’

Tet1 (mouse) 5’-ATTTCCGCATCTGGGAACCTG-3’

5’-GGAAGTTGATCTTTGGGGCAAT-3’

|  |
| --- |

**References**

Kanao, R., Hanaoka, F., and Masutani, C. (2009). A novel interaction between human DNA polymerase eta and MutLalpha. Biochem. Biophys. Res. Commun*. 3*89, 40–45.
